# Hypoxic Gene Signature of Primary and Metastatic Melanoma Cell Lines: Focusing on HIF-1β and NDRG-1

**DOI:** 10.4274/balkanmedj.galenos.2019.2019.3.145

**Published:** 2019-12-20

**Authors:** Mustafa Emre Ercin, Önder Bozdoğan, Tarık Çavuşoğlu, Nazan Bozdoğan, Pınar Atasoy, Mukadder Koçak

**Affiliations:** 1 Department of Pathology, Karadeniz Technical University School of Medicine, Trabzon, Turkey; 2 Clinic of Pathology, University of Health Sciences, Ankara Numune Training and Research Hospital, Ankara, Turkey; 3 Private Practice; 4 Clinic of Pathology, University of Health Sciences, Dr. Abdurrahman Yurtaslan Ankara Oncology Training and Research Hospital, Ankara, Turkey; 5 Department of Pathology, Kırıkkale University School of Medicine, Kırıkkale, Turkey; 6 Clinic of Dermatology, LÖSEV-LÖSANTE Children and Adult Hospital, Ankara, Turkey

**Keywords:** Hypoxia, hypoxia-inducible factor-1 beta, melanoma, N-myc downstream regulated gene-1

## Abstract

**Background:**

Hypoxia is an important microenvironmental factor significantly affecting tumor proliferation and progression. The importance of hypoxia is, however, not well known in oncogenesis of malignant melanoma.

**Aims:**

To evaluate the difference of hypoxic gene expression signatures in primary melanoma cell lines and metastatic melanoma cell lines and to find the expression changes of hypoxia-related genes in primary melanoma cell lines at experimental hypoxic conditions.

**Study Design:**

Cell study.

**Methods:**

The mRNA expression levels of hypoxia-related genes in primary melanoma cell lines and metastatic melanoma cell lines and at experimental hypoxic conditions in primary melanoma cell lines were evaluated by using real-time polymerase chain reaction. Depending on the experimental data, we focused on two genes/proteins, the hypoxia-inducible factor-1 beta and the N-myc downstream regulated gene-1. The expression levels of the two proteins were investigated by immunohistochemistry methods in 16 primary and metastatic melanomas, 10 intradermal nevi, and a commercial tissue array comprised of 208 cores including 192 primary and metastatic malignant melanomas.

**Results:**

The real-time polymerase chain reaction study showed that hypoxic gene expression signature was different between metastatic melanoma cell lines and primary melanoma cell lines. Hypoxic experimental conditions significantly affected the hypoxic gene expression signature. In immunohistochemical study, N-myc downstream regulated gene-1 expression was found to be lower in primary cutaneous melanoma compared to in intradermal nevi (p=0.001). In contrast, the cytoplasmic expression of hypoxia-inducible factor-1 beta was higher in primary cutaneous melanoma than in intradermal nevi (p=0.001). We also detected medium/strong significant correlations between the two proteins studied in the study groups.

**Conclusion:**

Hypoxic response consists of closely related proteins in more complex pathways. These findings will shed light on hypoxic processes in melanoma and unlock a Pandora’s box for development of new therapeutic strategies.

Melanomas are life-threatening malignant neoplasms originating from the pigment-producing cells derived from the pluripotent neural-crest tissue (1,2). Cutaneous melanoma is the most common type of primary melanoma encountered in clinical practice. The mucosal and ocular melanomas are relatively rare (3,4,5).

There has been an enormous effort to establish the molecular mechanisms involved in melanoma progression due to a significant increase in its incidence worldwide and a low chance of success in treatment of advanced stages (6,7). Molecular pathogenesis of melanoma is very complicated, and thus is intensely investigated. Besides the cellular and molecular mechanisms, it has been well documented that tumor microenvironment has a crucial role in oncogenesis and progression of melanoma (8,9).

Hypoxia is one of the well-defined and important microenvironmental factors affecting tumor proliferation, progression, and metastasis. Decreasing physiological oxygen levels, due to rapid proliferation and high metabolic demands of the tumor, is one of the characteristic features of solid tumors and is called as tumor hypoxia (10,11,12,13). There has been strong support that oncogenic pathways are activated by hypoxia in melanoma progression (14).

Major conductor of the hypoxia signaling orchestra is the hypoxia-inducible factor (HIF), which is composed of HIF-1α and HIF-1β/aryl hydrocarbon receptor nuclear translocator subunits (15,16). Under normoxic conditions, HIF-1α is degraded by the ubiquitin-dependent processes. However, in severe hypoxic conditions, HIF-1α accumulates in the cytoplasm and then translocates into the nucleus to heterodimerize with the β subunit (17,18). This, in turn, causes an increase in the expression of the hypoxia-regulated target genes (19).

In a metastatic environment, tumor cells may exhibit distinct gene expression profiles as compared to primary tumors. This might be related to the different microenvironmental conditions or the differences of the metastatic clones from the primary tumors. Metastatic tumor cells are commonly subjected to hypoxic conditions more than the primary site and, thus, need further genetic, epigenetic, and post-translational survival measures (20,21).

In this study, we aimed to evaluate the difference in hypoxia-related gene expression signatures in primary melanoma cell lines (PMCL) and metastatic melanoma cell lines (MMCL). We selected WM-115 and WM-266-4 cell lines, respectively, as PMCL and MMCL, originated from same patient. We also targeted to find the expression changes of hypoxia-related genes in PMCL at experimental hypoxic conditions. Depending on the current literature, the observed differences in gene expression patterns, and pathway analysis, we focused on two genes HIF-1β and N-myc downstream regulated gene-1 (NDRG-1). As a last step, we studied the immunohistochemical expression levels of the two selected proteins in nevus, primary, and metastatic human melanoma tissues.

## MATERIALS AND METHODS

### Study design

This study was designed as three steps. As a first step, we evaluated the differences of hypoxia-related gene expression patterns in PMCL (WM-115) and MMCL (WM-266-4). Then, we investigated the differences in mRNA expression levels of these genes of PMCL in experimental hypoxic conditions. As a last step, we focused on two proteins (HIF-1β and NDRG-1), which were found to change significantly in experimental studies. We established the distribution and differential expression patterns of the two proteins in nevus, primary, and metastatic melanomas.

### Archival materials

Thirteen primary malignant melanomas (7 males and 6 females) including two mucosal and one uveal melanoma, 3 metastatic melanomas (1 male and 2 females), and 10 intradermal nevi (1 male and 9 females) were included in this study. Mean age was 61.3 years in the melanoma group and 34.3 years in the intradermal nevi group. Clinical and histopathological features of archival materials are demonstrated in Supplementary Table 1. Since the archived pathology specimens were placed in formalin immediately after procurement, the cold ischemia time was kept minimum.

### Tissue array

Commercial tissue array ME2082 (US Biomax, USA) was comprised of 208 formalin-fixed, paraffin-embedded human tissues including 128 primary malignant melanomas, 64 metastatic malignant melanomas, and 16 normal skin tissues. Sixty-five of the 128 primary malignant cores were cutaneous melanomas, 13 were ocular, 39 were mucosal, and 11 were primary melanomas not otherwise specified (melanoma, NOS) localized in the soft tissues. Clinical and histopathological features of tissue arrays are listed in Supplementary Table 2.

### Cell culture

Both cell lines were procured from the American Type Culture Collection. Cell lines were cultured in Eagle's minimum essential medium (Lonza Verviers, Belgium) supplemented with heat inactivated 10% fetal bovine serum and 1% penicillin-streptomycin (v/v) in a humidified incubator at 34 °C (for WM-115) or 37 °C (for WM-266-4) in 5% CO_2_ environment.

### Hypoxia experiment

Hypoxic environment was achieved using BD GasPak EZ Gas Generating Container Systems and GasPak EZ Anaerobe Container System Sachets (Becton Dickinson and Co., Cockeysville, MD., USA). This system produces an anaerobic atmosphere within 2.5 h with less than 1.0% oxygen. PMCL were harvested after being exposed to 1, 4, and 8 h of hypoxia, and pellets of these cells were prepared for quantitative real-time polymerase chain reaction (qRT-PCR) studies and immunohistochemical analyses.

### RNA purification and cDNA synthesis

RNeasy Mini Kit (Qiagen, Hilden, Germany) was used to extract total cellular RNA from PMCL and MMCL. cDNA was synthesized from 2 µg of total RNA using RT^2^ First Strand Kit (Qiagen, Hilden, Germany).

### Real-time polymerase chain reaction

**Figure 1:**
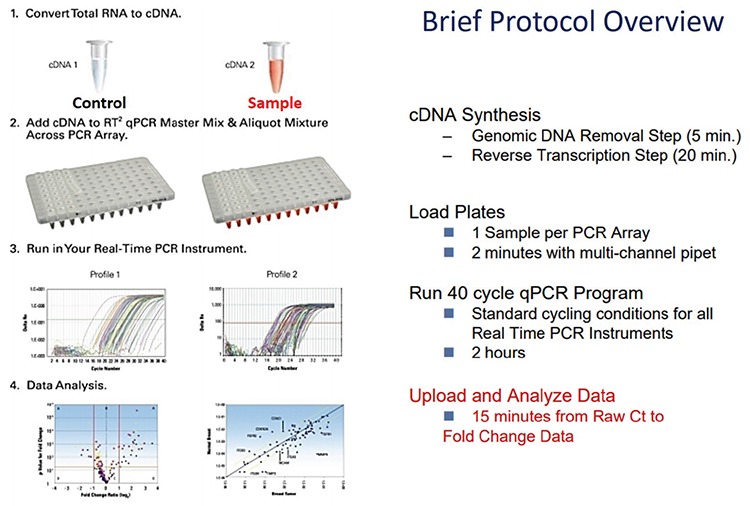
Protocol overview of RT^2^ profiler PCR array. PCR: polymerase chain reaction; RT: real-time

PCR studies were performed with the RT^2^ profiler PCR array (PAHS-032Z-Human Hypoxia Signaling Pathway PCR Array, Qiagen, Hilden, Germany) with RT^2^ Real Time SYBR Green PCR Master Mix in Rotorgene thermocycler (Qiagen, Hilden, Germany) (Figure 1). qPCR experiments were duplicated.

### Gene expression analysis

Expression levels of a spectrum of 84 hypoxia-related genes were determined via qRT-PCR and were analyzed using the Web-Based PCR Array Data Analysis software available on SABiosciences website. Expression values of all the samples were normalized to their relative expression levels of 5 housekeeping genes (ACTB, B2M, GAPDH, HPRT1, and RPLP0). A p value <0.05 was assumed statistically significant and the cut-off Ct value was selected to be 33 cycles. The differentially expressed genes were also analyzed by web-based gene analyses tool kit (WebGestalt- http://www.webgestalt.org/).

### Immunohistochemical staining

Immunohistochemical studies of archival cases and tissue microarrays were performed automatically in the Bond Max equipment (Leica Microsystems Inc., Wetzlar, Germany). Antigen retrieval steps were performed in Bond-Epitope Retrieval Solution 1 (AR9961) for HIF-1β antibody (Abcam, 1:200) and in Bond-Epitope Retrieval Solution 2 (AR9640) for NDRG-1 antibody (Santa Cruz Biotech, 1:300), at 100 °C. Detection was carried out with Bond Polymer Refine Red Detection kit (DS9390). Stained slides were dehydrated and covered with mounting medium (DAKO; s3023) and cover-slips.

### Immunohistochemical scoring

Stained tissue microarrays and histological slides were digitally imaged under 100x magnification using the Nikon Eclipse Ni-U and Nikon’s NIS-Elements D Microscope Imaging Software Version 4.0 (Tokyo, Japan). Digital images were evaluated using the H-score method with minor modifications (22). A simple MS Excel macro file was generated to calculate the H-scores (range 0-300) using the following equation. H-score = ∑ (*Pi x i*), where *Pi* coefficient is the percentage of stained cells and i is the intensity of staining [3+ (strong), 2+ (moderate), 1+ (weak), and 0 (absent) intensity].

### Statistical analysis

Statistical analysis was performed using PASW statistics v.17.0 (Chicago, IL, USA). Data were subjected to analysis of normality distribution using Shapiro-Wilk test. The differences between the H-scores of the groups were studied with the non-parametric Kruskal-Wallis one-way analysis test and then Mann-Whitney U test. Bonferroni correction was also applied for reducing the type I errors. The correlation between the parameters was investigated by Spearman's correlation.

### Ethics Statement

This study was ratified by the Ethics Committee of Kırıkkale University (26.04.2012 decision no: 12/175). Written informed consent was obtained from all the patients.

## RESULTS

### Real-time polymerase chain reaction

In this study, we aimed to show the expression differences of hypoxia-related genes in PMCL and MMCL. As a result, 37 genes were altered more than 2-folds: 10 genes were upregulated, while 27 genes were downregulated in the MMCL (Table 1). WebGestalt Pathway Commons analysis showed the differently expressed genes involved in several important pathways demonstrated in Table 2. Relying on the qRT-PCR results, pathway analysis, and the available literature, we selected HIF-1β and NDRG-1 genes for further investigation using immunohistochemical studies. HIF-1β, which is the second component of HIF complex, showed 7,8-fold upregulation in MMCL. NDRG-1 is an interesting protein, which has numerous functions including metastasis suppression and hypoxia-responsive properties. NDRG-1 showed 8,7-fold downregulation in MMCL. Both genes were also a part of all related pathways.

### Hypoxia

We analyzed the gene expression differences in PMCL after exposure to 1, 4, and 8 h of hypoxia (23). Seventy-four of the studied 84 genes were significantly changed after being exposed to 4 h of hypoxia. However, only 31 of the genes were still affected at 8 h (Table 3). The gene profile at 8 h was analyzed by WebGestalt Pathway Commons. The related pathways are demonstrated in Table 4. mRNA expressions of our candidate genes, HIF-1β and NDRG-1, were upregulated after 4 h of hypoxia. However, NDRG-1 gene was then significantly downregulated after 8 h. Immunohistochemical staining showed that NDRG-1_nuc_ was decreased, while HIF-1β_cyt-nuc_ and NDRG-1_cyt_ were increased in the PMCL after 1, 4, and 8 h of hypoxia.

### Immunohistochemical staining

**Figure 2:**
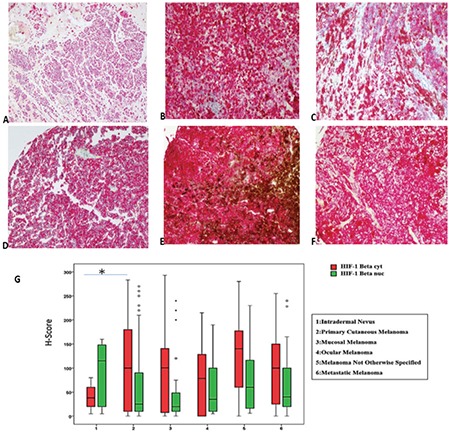
a-g. HIF-1β immunostaining in study groups, except for low intense positivity in intradermal nevus (a), significant positivity was detected in primary cutaneous (b, c), rectal (d), ocular (e), and metastatic melanoma (f). Original magnifications a; ×4; b; c; d; e; f, ×100. Box-plot graphs for HIF-1β immunostaining H-scores of study groups (g). HIF: hypoxia-inducible factor

**Figure 3:**
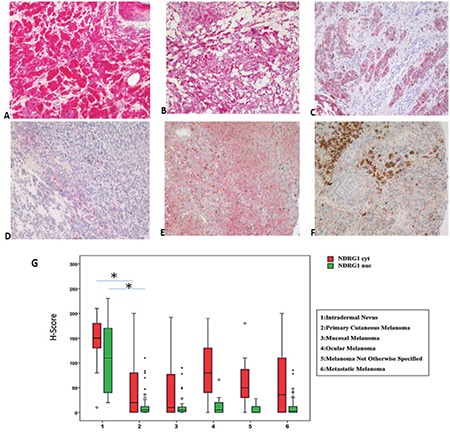
a-g. NDRG-1 immunostaining in study groups. Strong positivity is easily detected in intradermal nevus (a), weak and heterogenous positivity is detected in primary cutaneous (c), rectal (d), and metastatic melanoma (f). However, some primary melanomas (b) and ocular melanomas (e) show more intense positivity, though not as strong as in intradermal nevus. Nuclear and cytoplasmic scores of NDRG-1 in nevus are higher than melanomas (g). Original magnifications a, b, c ×200, d, e, f, ×100. Box-plot graphs for NDRG-1 immunostaining H-scores of study groups (g). NDRG-1: N-myc downstream regulated gene-1

Two markers showed both cytoplasmic (_-cyt_) and nuclear (_-nuc_) positivity. HIF-1β showed weak staining in intradermal nevus. However, in melanoma groups, significant positivity was detected (Figure 2). In intradermal nevus, NDRG-1 staining was both strong and homogeneous. Although staining was weaker and more heterogeneous in melanoma, some primary melanomas and ocular melanomas showed more intense positivity, though not strong and diffuse as in intradermal nevus (Figure 3). Quantitative immunohistochemical staining results (H-scores) of the study groups are demonstrated in boxplots in Figure 3.

In cell lines, NDRG-1_nuc_ staining was decreased in the MMCL, but HIF-1β_cyt-nuc_ and NDRG-1_cyt_ staining were increased.

### Statistical comparison

NDRG-1_cyt-nuc_ was found to be significantly lower in primary cutaneous melanoma tissues compared to in intradermal nevi (p=0.001). In contrast, HIF-1β_cyt_ was significantly higher in primary cutaneous melanoma than in intradermal nevi (p=0.001). There was no statistically significant H-score difference in cytoplasmic-nuclear staining between primary and metastatic melanomas for the two proteins.

A positive correlation was observed between NDRG-1_nuc_ and HIF-1β_nuc_ in intradermal nevi group (r=0.758; p=0.011).

## DISCUSSION

During carcinogenesis, neoplastic cells face different challenging conditions including hypoxia. However, neoplastic cells usually have the capacity to adapt with the hypoxic microenvironment, owing to various intracellular mechanisms. HIF-1 pathway is probably at the center of this adaptive hypoxic response in neoplastic cells (24,25). Activation of this pathway results in overexpression of several other important proteins having crucial roles in carcinogenesis and metastasis (11).

In this study, two established cell lines derived from the primary and metastatic melanomas of the same patients were used to reveal the differences in hypoxic gene expression. qRT-PCR analyses of the PCR array, which included 84 hypoxia-related genes, showed that the expression levels of 10 genes were upregulated, while of 27 genes were downregulated in the MMCL. The expression profile of the studied genes was related to several important pathways. It was reported that the expression levels of 576 genes were significantly different between primary and metastatic melanomas, and of these, 402 genes associated with cell cycle regulation, cell adhesion, protease inhibitor activity, and keratinocyte-related functions were downregulated in metastatic melanoma. It was shown that hypoxic microenviroment induces a more migratory and invasive cell type in malignant melanoma (26,27). In our study, the pathway analysis showed that besides expected HIF-1α pathways, important cancer progression, angiogenesis, and metastasis-related pathways were changed in MCCL. Our data showed that MCCL gained different gene signature than PMCL, basically on angiogenesis and migratory properties. It is well known that the affected pathways in our study; the platelet-derived growth factor receptor, vascular endothelial growth factor-vascular endothelial growth factor receptor, integrin family, focal adhesion kinase, and EGFR-related pathways; are closely related to invasion and metastasis in cancer. The vascular endothelial growth factor-vascular endothelial growth factor receptor, platelet-derived growth factor, and endothelial growth factor receptor pathways also have important roles in tumor angiogenesis (28,29). Insulin-like growth factor-1 and PI3K signaling are other well known pathways that are significant contributors in carcinogenesis (30,31). Glypican 1, an important protein at the center of the GP1 pathway, has significant roles in tumor growth, angiogenesis, and metastasis (32,33).

Similar to our study, it was observed that angiogenesis, invasion, and apoptosis-related genes were upregulated and tumor suppressor genes were downregulated in metastatic melanoma (34). HIF1-α-related hypoxic pathways are also changed in MMCL, and these data may show that these pathways are also important in metastasis. Hypoxia was especially important in melanoma progression and hypoxia-related genes were significantly upregulated in melanoma (34,35). It has also been emphasized that hypoxia response pathways are required for metastasis (36). Our findings support the previous studies and we speculate that hypoxia-related gene expression profiles may be significantly important in metastatic melanoma clones.

We also investigated the hypoxia responses of 84 hypoxia-related genes in PMCL under an experimental hypoxic condition. Seventy-four out of 84 genes were changed after 4 h of hypoxia, followed by a significant reduction to 31 genes in 8 h of hypoxia. At 8 hours, pathway analysis showed that affected pathways in PMCL, similar to MMCL, showed differentially expressed genes basically involved in progression, invasion, and metastasis. Furthermore, HIF-1α, insulin-like growth factor-1, and Glypican 1 pathways were also affected in hypoxia. The other affected pathways at 8 h of hypoxia, including CDC42 signaling, α9 β1 integrin signaling, and ILK signaling have also significant roles in invasion and metastasis (37,38). The mTOR signaling, which is one of the well known pathways in melanoma, controls cell growth, proliferation, and survival. Recent studies have shown that mTOR plays a critical role in the regulation of tumor cell motility, invasion, and cancer metastasis (39,40).

In this study, one of our selected genes/proteins, HIF-1β, showed higher expression levels in metastatic tumor cells and in hypoxic experimental conditions. HIF-1β has a critical role in hypoxic response (41). Contrary to initial findings, recent studies suggest that HIF-1β levels are not constant. Instead, its levels fluctuate in response to hypoxic conditions, akin to its heterodimeric partner HIF-1α (42). Similar to our results, hypoxic responses of aryl hydrocarbon receptor nuclear translocator was shown in melanoma cell lines (17,43). However, it was thought that this response was cell-type dependent. In tissue studies, we found HIF-1β to be significantly higher in primary cutaneous melanoma than in intradermal nevi. The information about its role in carcinogenesis is not well known. However, there are some clues involved in cell proliferation and survival of cancer cells (44).

Similar to HIF-1β, we found NDRG-1 levels to be upregulated in hypoxic conditions. There have been strong clues that NDRG-1 protein shows hypoxic responses. Upregulation of NDRG-1 in hypoxic conditions can be HIF-1-dependent, but was also postulated to be related to other mechanisms (45). Our studies showed that NDRG-1 mRNA expression was initially upregulated, and then was downregulated in experimental hypoxic conditions in melanoma cells. Immunohistochemical staining results showed a cytoplasmic expression of NDRG-1, but no nuclear expression, and was upregulated in PMCL after 1, 4, and 8 h of hypoxia. In the light of our data, it can be concluded that NDRG-1 is one of the possible genes that respond to hypoxia.

NDRG-1 mRNA levels were also downregulated in MMCL compared to in PMCL. However, at protein levels, only NDRG-1nuc staining was decreased in MMCL; whereas, the cytoplasmic H-scores were slightly increased. Although NDRG-1 was downregulated in melanoma compared to in nevus, we could not detect any difference between H-scores of primary and metastatic melanomas in tissue samples. It has been shown that NDRG-1 was downregulated in majority of cancers and acts as a metastasis suppressor protein in at least a group of human carcinomas (46,47,48). The importance of NDRG-1 in melanoma is not well known; strong NDRG-1 staining was seen in melanoma samples compared to in nevus. The discrepancy in these results and ours may be related to the differences in selected antibodies and cases, as well as the performed techniques (49).

In conclusion, we demonstrated that MMCL gain distinct gene expression signature compared to PMCL. Pathway analysis showed that this signature is involved in pathways related to invasion and metastasis besides hypoxic response. Interestingly, PMCL cells in hypoxic condition showed similar changes. We also demonstrated the HIF-1β and NDRG-1 expressions to be significantly different in nevus than in melanoma in paraffin-embedded tissue study. However, we could not find any difference in the two proteins between primary and metastatic melanomas. Our study, in the light of previous literature, showed that hypoxia is an important phenomenon and may contribute to melanomagenesis and metastasis of melanoma.

## Figures and Tables

**Table 1:**
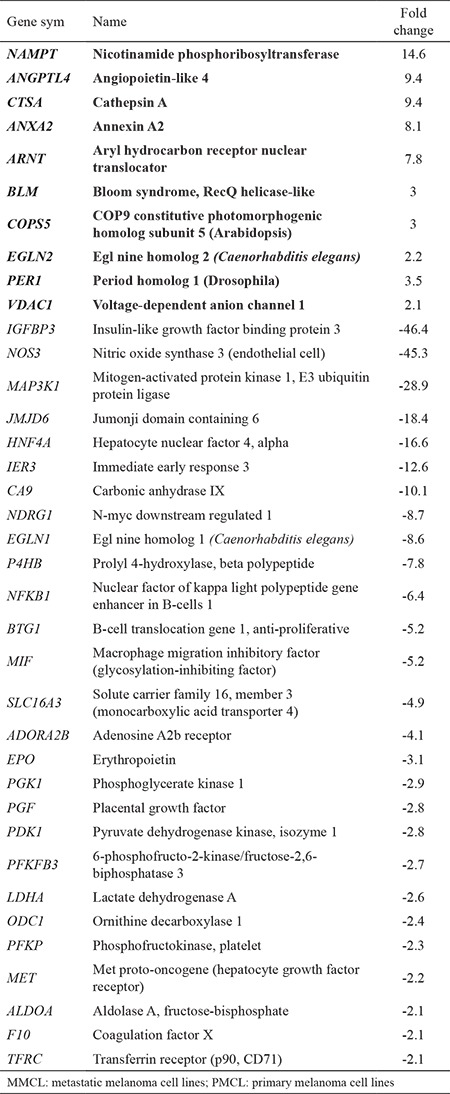
Differentially expressed genes of MMCL compared to PMCL

**Table 2:**
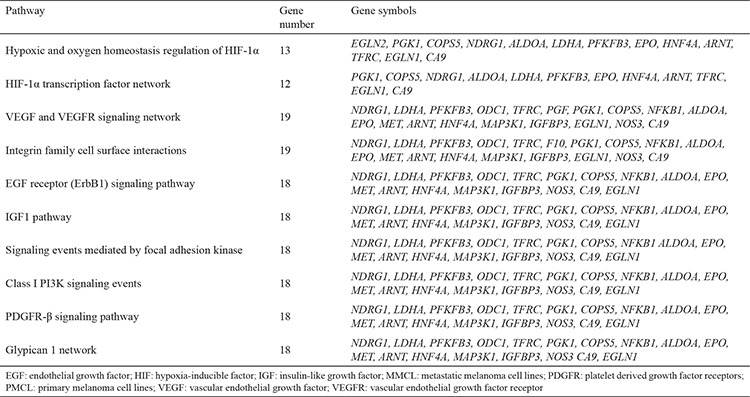
Pathways analysis of differentially expressed genes between MMCL and PMC

**Table 3:**
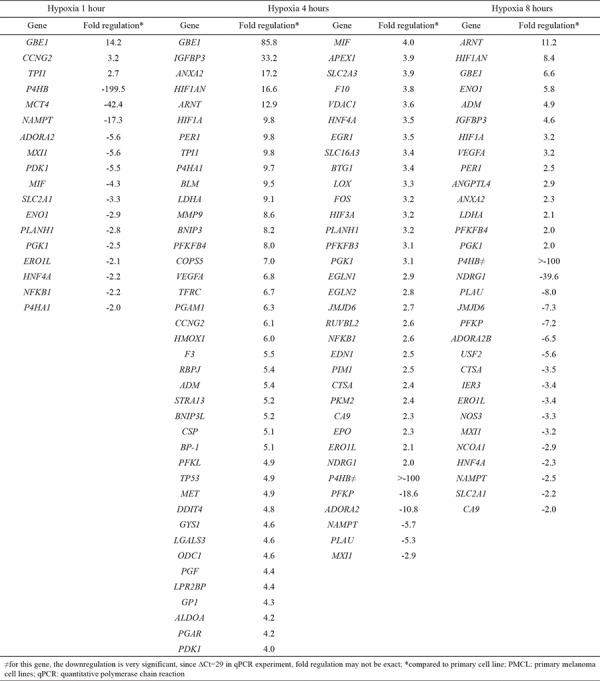
Fold changes of PMCL in experimental hypoxic conditions

**Table 4:**
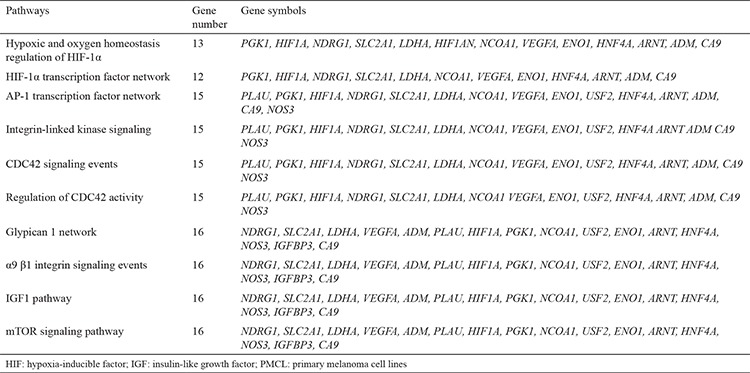
Pathways analysis of differentially expressed genes of PMCL at 8 hours hypoxia
